# Recent Advances in CRISPR/Cas9 Delivery Approaches for Therapeutic Gene Editing of Stem Cells

**DOI:** 10.1007/s12015-023-10585-3

**Published:** 2023-09-18

**Authors:** Malihe Lotfi, Dorsa Morshedi Rad, Samaneh Sharif Mashhadi, Atefeh Ashouri, Majid Mojarrad, Sina Mozaffari-Jovin, Shima Farrokhi, Maryam Hashemi, Marzieh Lotfi, Majid Ebrahimi Warkiani, Mohammad Reza Abbaszadegan

**Affiliations:** 1 Student Research Committee, Faculty of Medicine, Mashhad University of Medical Sciences, Mashhad, Iran; 2 Medical Genetics Research Center, Mashhad University of Medical Sciences, Mashhad, Iran; 3 School of Biomedical Engineering, University of Technology Sydney, Sydney, Australia; 4 Department of Medical Genetics and Molecular Medicine, Faculty of Medicine, Mashhad University of Medical Sciences, Mashhad, Iran; 5 Nanotechnology Research Center, Pharmaceutical Technology Institute, Mashhad University of Medical Sciences, Mashhad, Iran; 6 Department of Pharmaceutical Biotechnology, School of Pharmacy, Mashhad University of Medical Sciences, Mashhad, Iran; 7 Department of Medical Genetics, School of Medicine, Shahid Sadoughi University of Medical Sciences and Health Services, Yazd, Iran; 8 Institute for Biomedical Materials and Devices (IBMD), Faculty of Science, University of Technology Sydney, Sydney, Australia

**Keywords:** CRISPR/Cas9 system, Delivery technologies, Gene editing, Stem cells, Stem cell therapy

## Abstract

**Abstract:**

Rapid advancement in genome editing technologies has provided new promises for treating neoplasia, cardiovascular, neurodegenerative, and monogenic disorders. Recently, the clustered regularly interspaced short palindromic repeats (CRISPR)/CRISPR-associated protein 9 (Cas9) system has emerged as a powerful gene editing tool offering advantages, including high editing efficiency and low cost over the conventional approaches. Human pluripotent stem cells (hPSCs), with their great proliferation and differentiation potential into different cell types, have been exploited in stem cell-based therapy. The potential of hPSCs and the capabilities of CRISPR/Cas9 genome editing has been paradigm-shifting in medical genetics for over two decades. Since hPSCs are categorized as hard-to-transfect cells, there is a critical demand to develop an appropriate and effective approach for CRISPR/Cas9 delivery into these cells. This review focuses on various strategies for CRISPR/Cas9 delivery in stem cells.

**Graphical Abstract:**

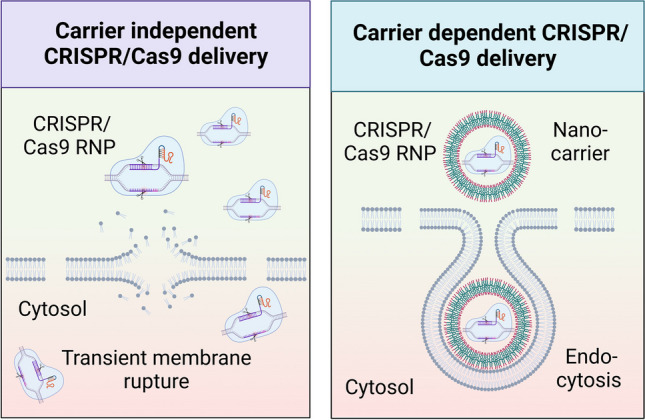

## Introduction

### Genome Editing and Programmable Nucleases

In recent years, advances in delivery technologies have enabled the intracellular loading of impermeable gene-editing tools, such as programmable nucleases, into different cell types. These technologies, coined as genome editing approaches, can lead to alterations in genomic DNA sequence through either insertion or deletion (indels) of one or more base pairs. Genome editing approaches have been exploited in various kinds of diseases, from treating life-threatening conditions (e.g., hereditary disorders) to restoring the lost function in gene expression studies [1, 2]. The genome-editing procedure mainly relies on creating double-strand breaks (DSBs) at the site of the target sequence. These site-specific DSBs can be repaired through two distinct endogenous pathways: i) non-homologous end-joining (NHEJ), which is the primary repairing pathway throughout the cell cycle, and ii) homology-directed repair (HDR). In NHEJ, protein factors force the DSBs to re-join and ligate the cleaved DNA strands without requiring a homologous template leading to the formation of indels followed by modification or inactivation of the target gene. With lower frequency, the HDR pathway can repair the DSBs only in the presence of an appropriate DNA donor template [3].

**Fig. 1:**
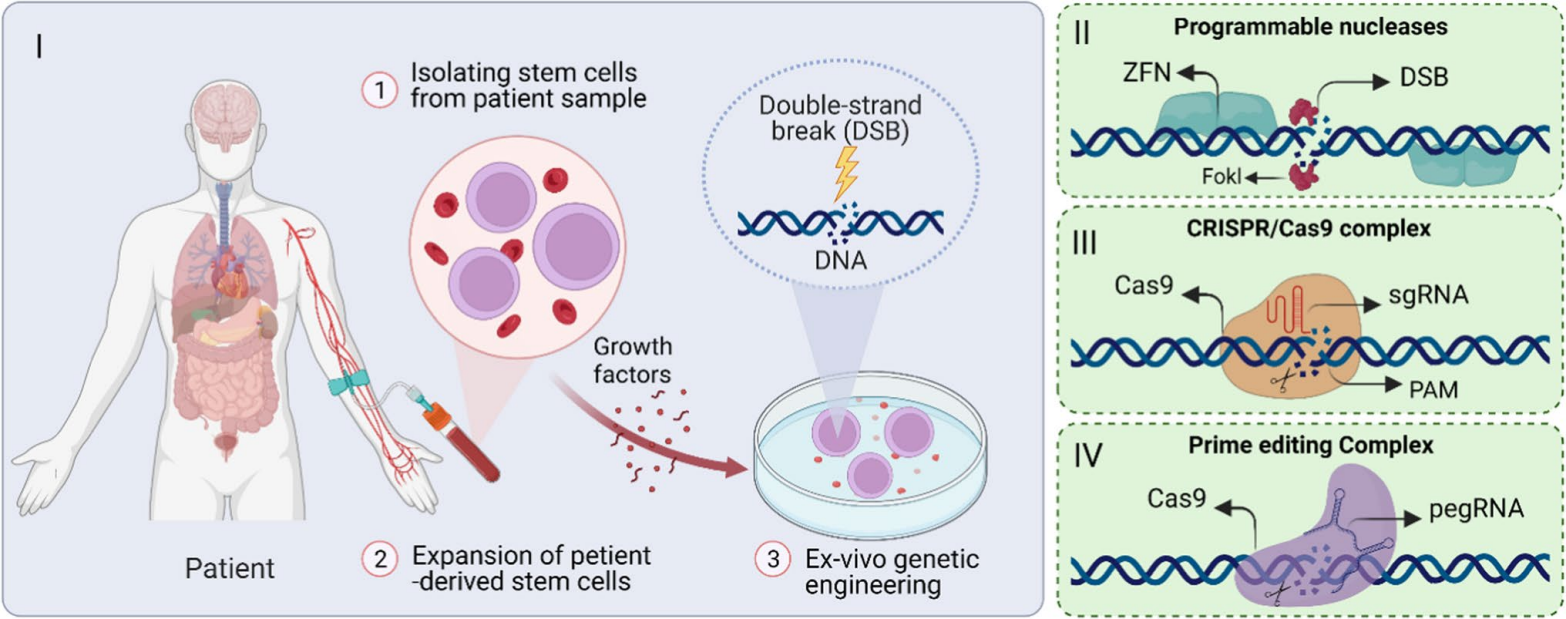
Schematic of the gene-editing procedure. Sample preparation process (I) and the leading gene-editing platforms, including ZFNs (II), CRISPR/Cas9 complexes (III), and prime editing complexes (IV). Sample preparation involves isolating stem cells from the patient blood sample, followed by expanding the isolated cells in culture. Next, ex vivo genetic engineering will take place, which involves creating double-strand breaks in DNA using programmable nucleases, CRISPR/Cas9 complex, or prime editing system

To date, different types of engineered nucleases, including meganucleases, transcription activator-like effector nucleases (TALENs), zinc-finger nucleases (ZFNs), CRISPR/Cas9, and prime editing complexes have been developed to generate DSBs at targeted DNA sequences [4] (Fig. 1). These engineered nucleases recognize the target sites through interactions of DNA-protein (meganucleases, TALENs, and ZFNs) or DNA-RNA (CRISPR/Cas9).

CRISPR/Cas9 is a novel gene-editing approach derived from the prokaryotic immune system produced in response to the exogenous nucleic acids of phages and plasmids. Since 2013, when Cong et al. employed CRISPR/Cas technology for efficient genome editing in eukaryotic cells, this approach has gained significant attention in the field [5]. Moreover, CRISPR/Cas editing tool has shown many advantages over the other engineered nucleases, such as i) guiding the nuclease to the targeted site through the simple base-pairing rules, ii) the possibility of synthesizing the guiding RNAs in vitro, and iii) generation of multiple DSBs synchronously by using different guide RNA sequences, which enables editing of several loci in the mammalian genome. Recent studies have employed CRISPR-Cas9 as the most popular gene editing approach to study cystic fibrosis [6, 7], sickle cell disease (SCD) [8, 9], Huntington’s chorea disorder [10, 11], Duchenne muscular dystrophy [12–14], chronic granulomatous disease [15, 16] retinitis pigmentosa [17] hemophilia [18, 19], and thalassemia [20–22].

The CRISPR/Cas9 system mainly comprises a nuclease, called Cas9, and a single guide RNA (sgRNA) containing a sequence-specific CRISPR RNA (crRNA), capable of recognizing 18–20 nucleotides of the target DNA, and an auxiliary trans-activating crRNA (tracrRNA) [23]. Currently, different systems are used in CRISPR/Cas9 mediated gene editing, including plasmid-based CRISPR/Cas9, ribonucleoprotein (RNP) complex-based Cas9 protein with sgRNA, and Cas9 mRNA with sgRNA [24].

In plasmid-based CRISPR/Cas9 gene editing, plasmid DNA encoding both customized sgRNA and Cas9 protein enters the nucleus to be transcribed into target sgRNA and Cas9 mRNA. Next, the Cas9 transcript is translated into Cas9 protein in the cytoplasm and then returned to the nucleus for genome editing [25]. Although CRISPR/Cas9 plasmid DNAs are more stable than CRISPR/Cas9 mRNA and RNP complexes, potential disadvantages attributed to this approach are the long-lasting expression of the CRISPR/Cas9 system, increased off-target effects, and the possibility of DNA integration into the host genome [26]. On the contrary, in mRNA-based CRISPR/Cas9 gene editing, the transcription of sgRNA and Cas9 mRNA is not required. Therefore, efficient gene editing can be performed faster while reducing the off-target effects. Upon cytoplasmic delivery of the sgRNA and Cas9 mRNA, the mRNA translation would take place to produce the Cas9 protein, followed by localization of both sgRNA and Cas9 protein to the nucleus for gene editing [27].

The RNP complex-based system (Cas9 protein and sgRNA) is the most efficient and rapid gene editing approach, in which neither transcription nor translation is required. Since the CRISPR/Cas9 RNP complex does not naturally exist in the cells of interest, this highly efficient approach offers minimal off-target effects. Despite these advantages, this approach suffers from the high cost of producing RNP complex and toxic side effects [28].

#### Stem Cell-Based Therapeutic Applications of CRISPR

**Fig. 2:**
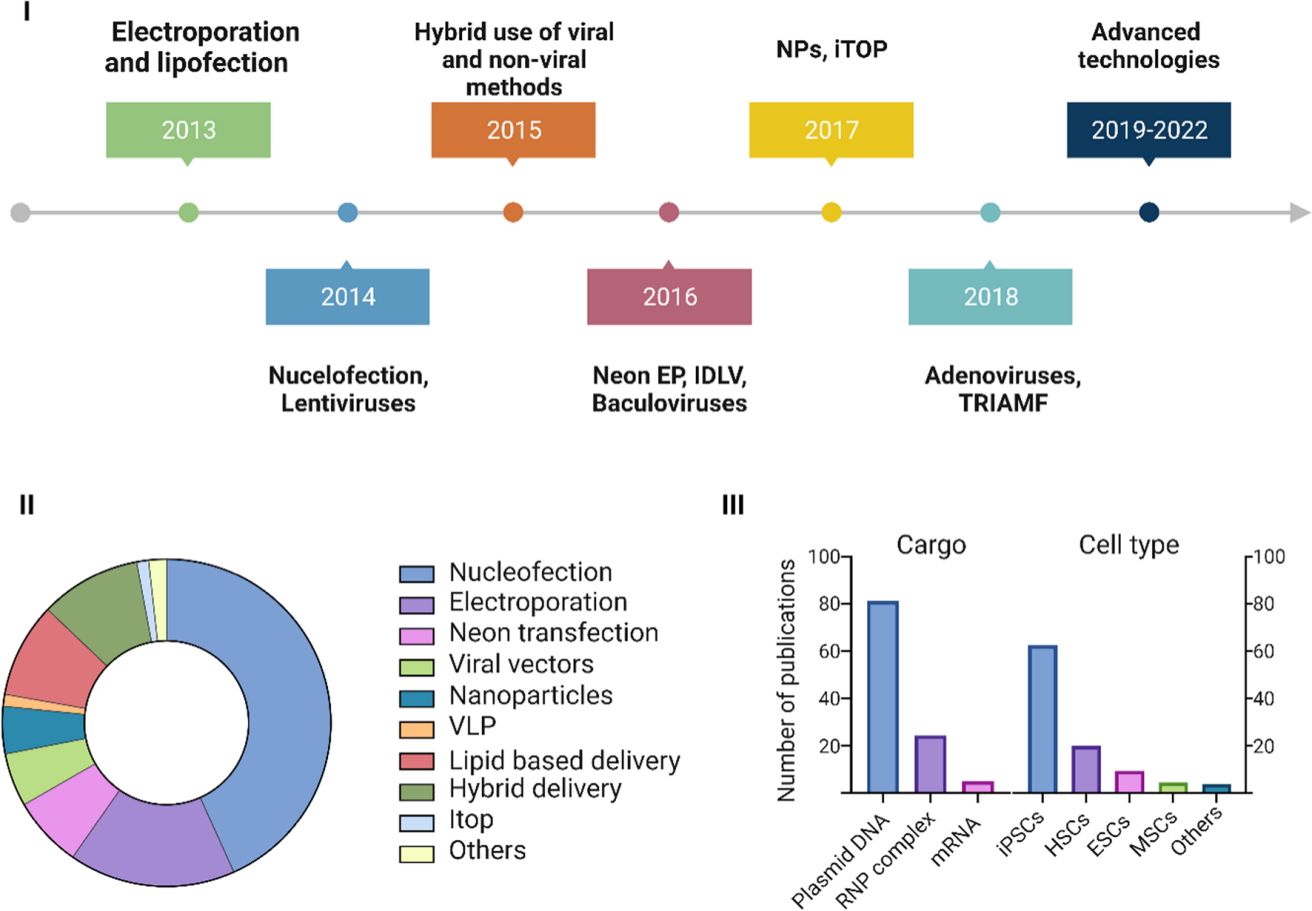
Research studies on the therapeutic applications of CRISPR/Cas9-mediated gene editing in stem cells. I) Since 2013, several delivery technologies have been used for cytoplasmic delivery of the CRISPR/Cas9 gene editing tools into the hPSCs. Different delivery techniques (II) have been used to load various cargoes into a wide range of cell types (III)

A variety of genetic disorders arise from mutations at the genomic level. Stem cell-based therapies have demonstrated efficacy in treating these diseases in clinical trials. This promising strategy contains the autologous transplantation of genetically edited stem cells. These genetically modified stem cells offer several advantages, such as self-renewal capacity, increasing the patient’s lifetime, and dividing into daughter cells with the ability to be differentiated into resident cells within different tissues [29]. Growing evidence indicated that genome editing through CRISPR/Cas9 system is an efficient approach extending to stem cell-based research and therapies. HPSCs have great proliferation potential and can differentiate into various cell types. These cells (e.g., neural, hematopoietic, and mesenchymal stem cells) can be manipulated using CRISPR/Cas9 gene editing tools and employed for therapeutic applications [30, 31]. An online search ranging from 2010 until 2022 (15/07/22) was performed on PubMed using the terms “CRISPR/Cas9” and “stem cell”. The search results revealed that several studies used the CRISPR/Cas9 system for the genetic engineering of the hPSCs (Fig. 2). Since the hPSCs are naturally impermanent to external cargoes such as CRISPR/Cas9 gene editing tools, these studies have used external forces for transient cell membrane disruption and cytoplasmic loading of these tools inside the cells, which will be discussed in the next section.

## Delivery Systems for Gene Editing by CRISPR/Cas9 in Stem Cells

**Fig. 3:**
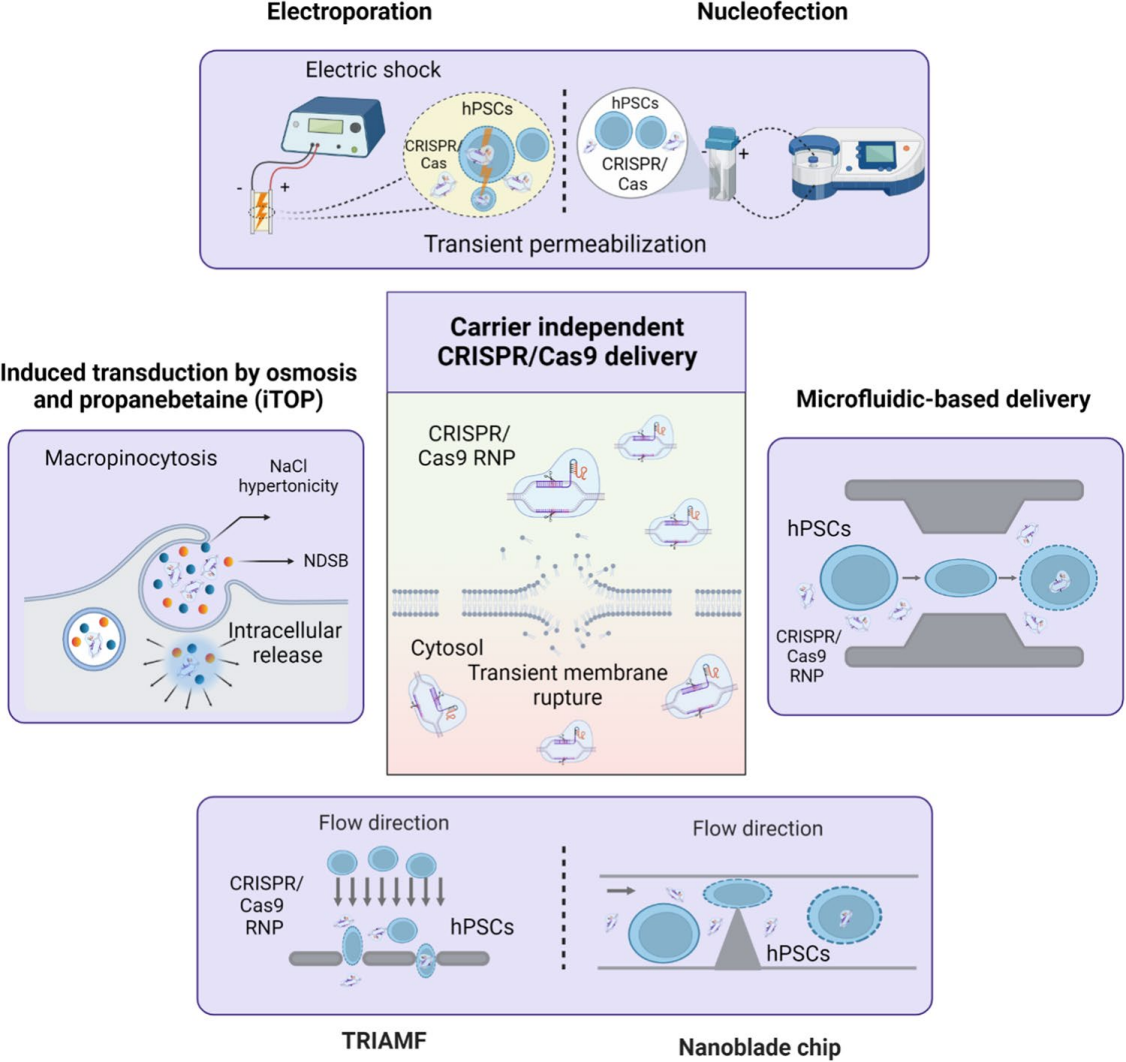
Carrier independent strategies for CRISPR/Cas9 delivery into stem cells. These approaches can be divided into physical methods, including electroporation and induced transduction by osmosis and propanebetaine (iTOP), and mechanical methods, such as microfluidic-, silicon nanoblade-, and transmembrane internalization assisted by membrane filtration (TRIAMF)-based delivery systems

**Fig. 4:**
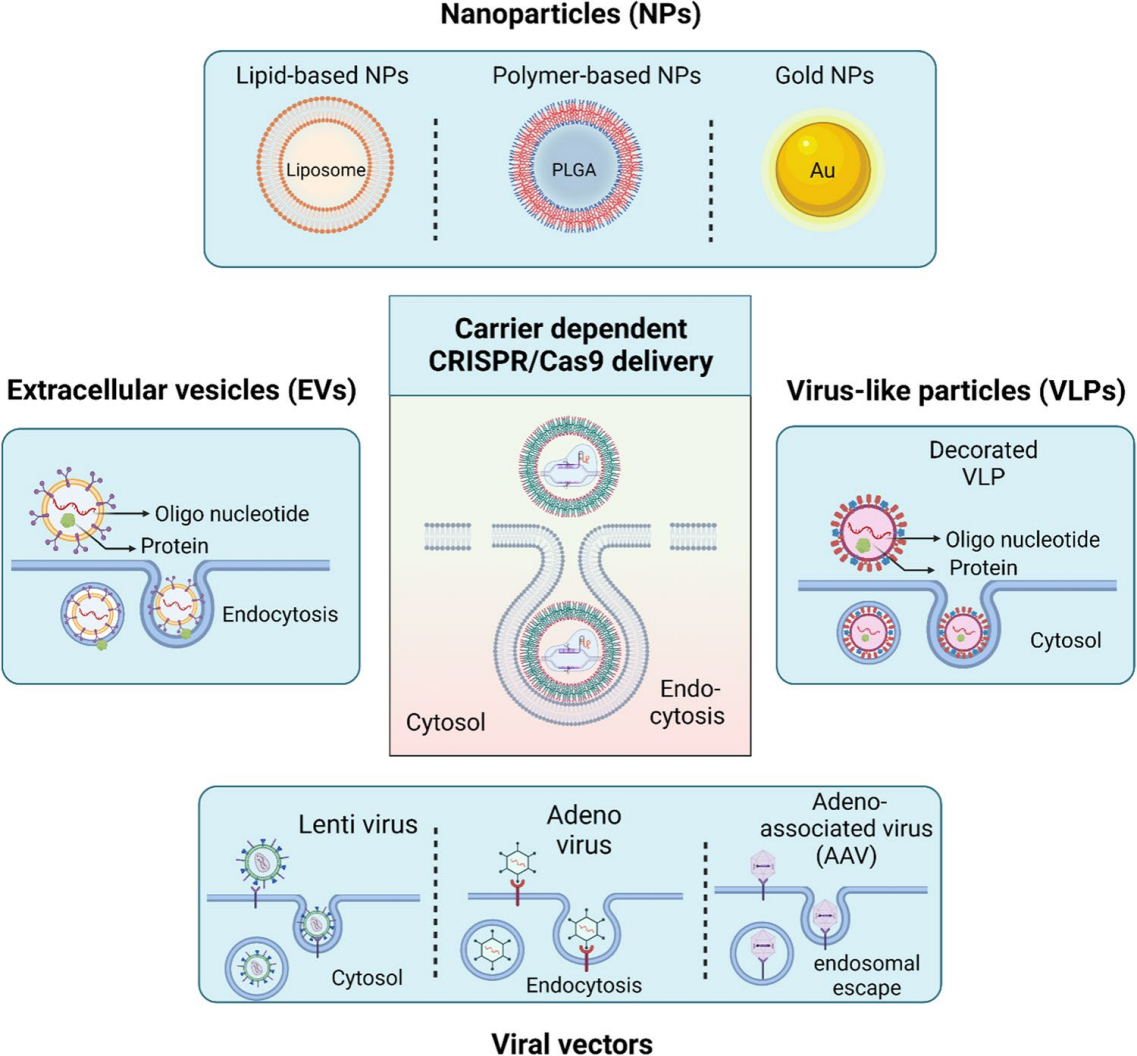
Carrier dependent CRISPR/Cas9 delivery strategies. These modalities are classified into nanoparticles (e.g., lipid, polymer, and gold), extracellular vesicles, viruses (e.g., lenti, adeno, and adeno-associated viruses), and viral-like particles

During the past decades, several delivery technologies have been developed to transiently permeabilize hPSCs plasma membrane, followed by facilitating the cytoplasmic delivery of the CRISPR/Cas9 genome editing tools. These technologies have successfully loaded the CRISPR/Cas9 tools inside the hard-to-transfect hPSCs for efficient genome editing with high specificity delivery efficiency [32]. This review focuses on carrier independent (e.g., physical and mechanical delivery) (Fig. 3) and dependent (e.g., nanoparticle, extracellular vesicles, viral-like particles, and viruses) strategies that have been employed to load CRISPR/Cas9 gene editing tools into different types of stem cells (Fig. 4).

### Carrier Independent Delivery Approaches

Carrier-independent approaches comprise physical and mechanical methods that utilize external forces to concurrently induce transient membrane disruptions and cargo delivery. These modalities, also coined as direct membrane permeabilization strategies, can create pores of different sizes allowing the passive diffusion of exogenous cargoes across the plasma membrane [33]. In this section, we will cover physical and mechanical delivery approaches that have been exploited for CRISPR/Cas delivery into the cells of interest.

#### Physical Delivery

Physical delivery methods are considered an effective approach for the direct delivery of nucleic acids, also called transfection, into the hard-to-transfect cells [34]. During the past decade, physical delivery approaches have been widely used for loading both CRISPR/Cas9 plasmid DNA and RNP into various cell types. The commonly used approaches for delivering CRISPR/Cas9 into different types of stem cells include electroporation, nucleofection, induced transduction by osmocytosis and propanebetaine (iTOP) [35], and mechanical transfection [36], which will be covered in the following sections. Although these approaches are simple and highly reproducible, challenges include processing a bulk population of cells resulting in heterogeneous responses within the cell population [37, 38].

##### Electroporation

Electroporation is a physical delivery approach that uses a series of controlled electric pulses to induce transient membrane permeabilization and cytosolic uptake of impermeable macromolecules such as plasmid DNA and gene constructs [39]. In this approach, a bulk population of cells is resuspended in an electroconductive buffer and added to a cuvette placed between two electrodes. Therefore, applying controlled electric pulses with optimized voltages and widths to the target cells can result in the creation of transient pores across the plasma membrane, facilitating the passive influx of the impermeable plasmid DNA [32]. Electroporation is considered as a highly efficient delivery approach that can increase the overall uptake and expression level of the exogenous DNA up to 1000 folds. It is worth noting that the delivery efficiency of this technique mainly relies on different parameters, including electric field characteristics (voltage, width, and number of pulses), electrode geometry, and cell and cargo types. One-size-fits-all gene delivery through electroporation has been elusive as optimal delivery conditions must be determined based on different cell and cargo types. Gene delivery using cuvette-based electroporation has shown promise in different applications, from vaccine development, transgenes expression, and enzyme replacement to cancer treatment. Since cuvette-based electroporation is a heterogeneous treatment that can induce different levels of stress in the target cells depending on their position relative to the electrodes, the clinical application of this conventional approach has been restricted [40]. Besides this, electroporation is a costly method that requires rather expensive reagents and kits, hindering its application in many laboratories.

Along with the technological advances in the field, significant efforts have been made toward implementing electroporation at the microscale. As a result, the Neon™ transfection system, which works by inducing an electric field within capillaries, has been developed and further commercialized by Invitrogen/Thermo fisher. This new user-friendly setup is more flexible and is designed to address the challenges of the conventional cuvette-based electroporation strategy. In this setup, the transfection will happen within a biologically compatible pipette tip chamber, which has been employed to generate more uniform electric fields. This technique maximizes the distance between electrodes while minimizing their surface area to ensure a controlled electric pulse is passed through the target cells to achieve a homogenous exposure and treatment. This rapid and highly reproducible technique has significantly improved the delivery outcomes in hard-to-transfect cells compared to the cuvette-based electroporation [41]. In 2019, this technology was used for the co-delivery of Cas9 protein, gRNA, and two indistinguishable donor DNA templates into induced pluripotent stem cells (iPSCs). This resulted in increased efficiency (~8.3%) of inducing in vitro homozygous modifications while maintaining cell viability [42].

In 2018, Xu et al. reported a tube-based electroporation method for efficient genome editing by delivering the CRISPR/Cas9 RNP into nearly 90% of the iPSCs without affecting the cell viability. As a result, a relatively high HDR rate (42.1%) was achieved in the edited cells, which is significantly higher than those reported in the previous studies within the range of 2.1–6.7% [43, 44]. In a recent study, Yudovich and colleagues performed viral-mediated delivery of sgRNAs into hematopoietic stem and progenitor cells (HSPCs), followed by Cas9 mRNA delivery using BTX ECM 830 electroporation. Using this combinational strategy, they could achieve up to 90% knockout of CD44 and CD45 cell surface proteins [45].

Nucleofection is another commercially available gene delivery strategy, which is mostly designed for the nuclear delivery of cargoes like siRNA, DNA, and oligonucleotides into hard-to-transfect and primary cell lines. This method is one of the most popular cuvette-based electroporation configurations that utilizes cell line-specific buffers along with nucleofector pulsing parameters for the direct delivery of nucleic acids into the nucleus. Nucleofection has become a popular protocol for the highly efficient delivery of CRISPR/Cas9 plasmids into different types of stem cells regardless of the cell cycle status [46–48]. Using the Amaxa Nucleofector 2 device (Lonza), Shinkuma et al. delivered CRISPR/Cas9 plasmid into the iPSCs to target a dominant negative mutation (c.8068_8084delinsGA) of COL7A1 gene in dominant dystrophic epidermolysis bullosa (DDEB). Using this strategy, they could achieve up to 90% editing efficiency through the NHEJ pathway in iPSCs generated from the DDEB patient’s fibroblasts [49]. In a follow-up study, Amaxa Nucleofector 2 device was used for co-transfection of spyFiCas9 and gRNA expression vectors into the iPSCs of recessive dystrophic epidermolysis bullosa (RDEB) patients. As a result, single and double allele mutation corrections in COL7A1 were achieved by activating the HDR pathway with efficiencies of 40% and 10%, respectively [50]. While HDR-mediated gene correction results in lower efficiencies, it is considered to be a more precise gene editing pathway than NHEJ. The lower efficiencies achieved through the HDR pathway can be attributed to the lower frequency of homologous recombination in target cells. Table 1 summarizes the studies that leveraged electroporation for CRISPR/Cas9 delivery, mostly in the form of CRISPR/Cas9 RNP, into different types of stem cells [136–138].

**Table 1:** Electroporation and nucleofection mediated delivery of CRISPR/Cas9 into the stem cells

Delivery approach	Stem cell type	DNA repair	CRISPR/Cas9 format	Donor format	Editing efficiency (%)	Reference
Electroporation-mediated CRISPR/Cas9 delivery	iPSCs	HDR	RNP	ssODN	20–70	[43, 51–53]
			mRNA	Plasmid DNA	2.3	[54]
			Plasmid DNA	ssODN	1–20	[44, 55–57]
				Plasmid DNA	4.2–43.5	[13, 58–62]
		NHEJ	Plasmid DNA	–	N/A	[18, 63]
		ABE	Plasmid DNA	–	24.5	[64]
	PSCs	HDR	mRNA	Plasmid DNA	11–17.1	[65]
		NHEJ	Plasmid DNA	–	2–34	[66]
	HSCs	HDR	mRNA	ssODN	21	[16]
				Plasmid DNA	9	[67]
				mRNA	8–23.5	[68]
			RNP	ssODN	N/A	[68]
		NHEJ	RNP	–	15	[69]
		MMEJ	RNP	–	75	[70]
		ABE	RNP/ mRNA	–	44/80	[71]
	ESCs	NHEJ	Plasmid DNA	–	–	[72]
Nucleofection-mediated CRISPR/Cas9 delivery	iPSCs	HDR	Plasmid DNA	Plasmid DNA	2–96.7	[7, 73–89]
				ssODN	0.7–78	[90–104]
				PCR product	2.8–3.1	[105]
				dsDNA	22	[106]
			RNP	ssODN	19–70	[50, 52, 53, 107, 108]
		NHEJ	Plasmid DNA	–	10–95	[49, 109–117]
			RNP	–	47–100	[118]
		ABE/CBE	mRNA	–	13–47	[119]
		Prime editor	mRNA	–	90	[119]
	HSCs	HDR	mRNA	ssODN	8	[120]
			RNP	Plasmid DNA	7.6–24.5	[121, 122]
				ssODN	25	[123]
		NHEJ	Plasmid DNA	–	42	[124, 125]
			RNP/ mRNA	–	2–83	[126–131]
		Base editor	RNP	–	45.5–69.1	[132]
	ESCs	NHEJ	Plasmid DNA	–	N/A	[133, 134]
			RNP	–	N/A	[34]
	MSCs	NHEJ	Plasmid DNA	–	53–77	[135]

##### Induced Transduction by Osmocytosis and Propanebetaine (iTOP)

iTOP is another carrier independent delivery strategy that utilizes a hyperosmolar buffer to load cargoes, such as proteins, into different cell types. This delivery buffer consists of propanebetaine and sodium chloride acting as a transduction compound to trigger macropinocytosis and non-specific internalization of the extracellular fluid through engulfment of the 0.5–5 μm vesicles. This approach has been used for efficient intracellular delivery of sgRNAs and Cas9 protein separately and together in the form of CRISPR/Cas9 RNP complex [139]. The iTOP method allows the loaded protein to transiently manipulate the cells and induce changes in cell function or epigenetic status. However, this approach has not gained much attraction due to its lower gene editing efficiency in primary cells compared with other commonly used delivery technologies [34, 140–143]. D’Astolfo and colleagues performed one and two consecutive rounds of iTOP to deliver CRISPR/Cas9 to human embryonic stem cells while achieving 10% and 26% gene editing efficiency, respectively [141]. Since iTOP leverages NaCl-mediated delivery of the Cas9 protein, it can induce damage to desired cells and delivery protein resuspended in a highly concentrated salty solution, making this strategy inapplicable for in vivo gene editing purposes.

#### Mechanical Delivery

Creating transient ruptures in the phospholipid bilayer can be achieved by inducing mechanical forces to the cells of interest. Mechanical delivery, coined as mechanoporation, has been performed through physical contact with a firm structure or exposure to fluid shear forces directed at the cell surface. These mechanisms will result in lipid heads’ instabilities, facilitating the transient pore formation and passive cargo transports [144]. One advantage of mechanical delivery is the efficient delivery of large-sized cargoes, including plasmid DNA, which is challenging using benchtop delivery options. Moreover, this approach has shown utility for the efficient delivery of genetic materials into hard-to-transfect immune and stem cells. In recent studies, mechanical delivery has been employed for CRISPR/Cas9 RNP complex delivery into HSPCs demonstrating the further applicability for ex vivo cell therapies [145, 146]. In 2017, Ma et al. developed a microfluidic chip named nano-blade chip (NB-Chip) for transient mechanical deformation and highly efficient delivery of the CRISPR/Cas9 complex into the CD^34+^ HSPCs. The proposed asymmetrical microchannel features a silicon nanoblade structure on one side of the deformation zone to induce contact pressure to the CD^34+^ HSPCs leading to membrane disruption. During the design optimization, the nano-blade structure stiffness and sharpness were substantially increased, which further assisted the efficient delivery of biomaterials. Furthermore, the treated HSPCs had long-term viability and maintained inherent multi-potency [145].

Another form of mechanical delivery is filtroporation which utilizes fluid shear forces to generate disruptions in the plasma membrane. This technique gained less attention until 2018 when transmembrane internalization assisted by membrane filtration (TRIAMF) was developed based on the filtroporation using track-etched membranes to load CRISPR/Cas9 complex targeting β2-microglobulin into CD^34+^ HPSCs. By forcing the cell suspension through the membrane’s micropores, fluid shear forces are generated, which further induce cellular stress to the point of creating transient membrane ruptures and, in turn, intracellular loading of cargo molecules to the target cells [146].

### Carrier Dependent Delivery Approaches

Carrier dependent delivery strategies rely on biological and chemical vectors to encapsulate the exogenous cargoes and bypass the plasma membrane barrier. Not only do these vectors protect the cargo from degradation but also, they facilitate the internalization of the cargo to the intended intracellular compartment. The cargo internalization mechanism for vectors is either through endocytosis or membrane fusion. This section will provide further details on different carrier dependent delivery systems, including nanoparticles, extracellular vesicles, virus-like particles, and viral vectors (Fig. 4).

#### Nanoparticle Delivery

In recent years, nanoparticles (NPs) have been widely used as promising carriers of the CRISPR/Cas9 complex, attracting great attention. Remarkable advances in nanoparticle research have revolutionized the field of controlled therapeutic delivery due to the advantages, including high efficiency, low cost, non-immunogenicity, and non-mutagenicity. To date, different types of lipid- and polymer-based as well as gold NPs have been developed and used for CRISPR/Cas9 delivery into different cell types [147]. In the following sections, we will provide an overview of the mechanism of action and application of these NPs in CRISPR/Cas9 delivery research.

##### Lipid-Based NPs (LNPs)

Lipid-based NPs, also known as liposomes, are spherical vesicles made up of phospholipid bilayers acting as effective delivery vehicles in different biological applications, including intracellular delivery of genetic materials into the target cells and treatment of different diseases in clinical practice [148]. Since plasma membrane and nucleic acids both feature negative charges, the electrostatic repulsion hinders the entrance of exogenous nucleic acids into the cells of interest. To overcome this challenge, nucleic acids are encapsulated into positively charged liposomes facilitating the cargo delivery and subsequent cellular uptake by converting the repulsive to attractive electrostatic forces [149]. LNP-mediated CRISPR/Cas9 delivery is a food and drug administration (FDA) approved strategy, which induces less stress to the desired cells leading to higher cell viability than its counterparts. However, this modality suffers from low delivery efficiency as it mainly relies on the endosomal pathway for cargo internalization [150, 151].

Advances in nanotechnology have enabled the development of the lipofectamine reagent, which is now the first preferred option for LNP-mediated delivery [150]. In a study, Lipofectamine® 3000 has been used as a CRISPR/Cas9 delivery tool for correction of the suspected causative SCN5A variant (rs397514446) in iPSCs-derived cardiomyocytes of patients with Brugada syndrome (BrS) [152]. Further studies with a focus on gene editing through lipofectamine-mediated delivery of CRISPR/Cas9 system into stem cells are summarized in Table 2.

**Table 2:** Carrier dependent techniques for CRISPR/Cas9 delivery into the stem cells

Delivery approach	Stem cell type	DNA repair	CRISPR/Cas9 format	Donor format	Editing efficiency (%)	Reference
Nanodiamonds	iPSCs	HDR	Plasmid DNA	linearized DNA construct	19.3	[153]
NanoMEDIC vesicle	iPSCs	Exon skipping	RNP	N/A	Up to 92	[154]
Gold nanoparticle	ESCs, iPSCs	HDR	RNP	Plasmid DNA	N/A	[155]
Colloidal gold nanoparticles	HSCs	HDR	RNP	ssDNA	7.8–8.1	[156]
Nano-silicon-blade	iPSCs, HSCs	NHEJ	RNP	N/A	50–82	[145, 157]
Exosome-liposome hybrid nanoparticle	MSCs	NHEJ	Plasmid DNA	N/A	N/A	[158]
PLGA-nanoparticles	HSPCs	NHEJ	RNP	N/A	38.4	[69]
VEsiCas VLP	iPSCs	NHEJ	RNP	N/A	17	[159]
Lipofectamine	iPSCs	HDR	Plasmid DNA	Plasmid DNA	N/A	[152]
				ssODN	N/A	[160]
		NHEJ	Plasmid DNA	N/A	N/A	[161]
		Base editing	N/A	N/A	21–37	[162]
	ESCs, iPSCs, ESCs, MSCs, and intestinal stem cells	NHEJ	Plasmid DNA	N/A	N/A	[6, 163–167]
	MSCs	CRISPRa	Plasmid DNA	N/A	N/A	[168]

##### Polymer-Based NPs

In recent studies, cationic polymer-based NPs have been widely used for different purposes, including gene delivery. These NPs can form polyplexes containing nucleic acids (nucleic acid/polycation complexes) through electrostatic interactions between the cationic group of the NPs and negatively charged nucleic acids [169, 170]. Polymer-based NPs can be formulated with different copolymer compositions and molecular weights with various degradation times ranging between several months to years [171, 172]. These NPs have been reported to improve the carrier-mediated delivery of CRISPR/Cas9 components [173]. Among the polymer-based NPs employed for CRISPR/Cas9 delivery is poly lactic-co-glycolic acid (PLGA) which is a biodegradable polymer as its hydrolysis can result in the formation of glycolic acid, lactic acid, and metabolite monomers. Since glycolic and lactic acids are both endogenous and can be readily metabolized through the Krebs cycle inside the human body, using PLGA-based NPs for biological applications and drug delivery purposes has shown minimal cytotoxic effects [174]. Owing to the advantages of PLGA-based NPs, using these NPs for drug delivery applications into the human body has been approved by the US FDA and the European medicine agency (EMA).

In a recent study, PLGA-based NPs were designed to encapsulate Cas9 protein (*S. pyogenes*) and gRNA and used as a carrier for CRISPR/Cas9 delivery into HSPCs. Upon successful delivery of the CRISPR/Cas9 complex, a rapid release of gRNA and Cas9 protein was observed, which was subsequently replaced with a continuous cargo release pattern resulting from endosomal/lysosomal escape and cytosolic penetration. More importantly, Cruz et al. demonstrated that PLGA-based NP-mediated gene-editing of HSPCs using CRISPR/Cas9 complex did not induce cellular cytotoxicity. Upon escaping from the lysosomal compartments, CRISPR/Cas9-PLGA-based NPs could efficiently (up to 40%) edit the γ-globin gene locus resulting in a significant increase in the expression level of fetal hemoglobin in primary erythroid cells [175]. Besides the advantages offered by these NPs, size dissimilarities and unpredictable behavior and interaction of NPs with target cells are the remaining challenges of the field which require further investigations.

##### Gold NPs (au NPs)

Along with the advances in nanotechnologies, inorganic NPs like Au NPs and magnetic NPs were developed and employed as appropriate carriers for gene delivery applications. Among inorganic NPs, Au NPs hold the potential to be used as multifunctional gene delivery systems due to their simple synthesis and modification process, high loading capacity, high cellular uptake, and inherent biocompatibility [176, 177]. Since these NPs are chemically inert, Au NP-mediated cargo delivery usually does not induce adverse immune responses inside the body [178]. Despite these advantages, Au NPs can induce cytotoxic effects at high concentrations eliminating their applications in clinical settings. However, a large number of studies have reported the use of Au NPs for CRISPR/Cas9 RNP complex delivery in both in vivo and in vitro conditions [155, 177, 179]. In a study, Au-NP-CRISPR/Cas9 carriers were generated through multiple formulation steps. First, Au NPs with the size of 15 nm were conjugated to single-stranded DNA sequences (50 thiols modified) that were hybridized to single-stranded donor DNA and generated the Au-NP-Donor complex. Then, CRISPR/Cas9 RNPs were loaded on Au-NP-Donor complex and further coated with silica and PAsp (DET) polymer to escape the endosomal entrapment resulting in cytosolic cargo release. The resulting Au-NP-Donor-CRISPR/Cas9-silicate carriers could successfully induce HDR in primary cells and cell lines in vitro with an editing efficiency of about 4% which is higher than lipofectamine-mediated transfection and nucleofection. Furthermore, muscular injection of NP-Donor-CRISPR/Cas9-silicate carriers in four-week-old *mdx* mice resulted in vivo correction of a point mutation in the dystrophin gene through the HDR pathway with editing efficiency of about 5.4% [155].

In another study, a CRISPR/Cas9 delivery system was developed by nano-formulation of colloidal Au NPs with the ability to enter the cells without the aid of external forces. The final monodispersed Au NP/crRNA nanoconjugates avoided lysosomal entrapment and were localized in the nucleus of HSPCs without inducing cytotoxicity. Genome editing at different points of interest in HSPCs was successfully achieved using these NPs nano-conjugated with different CRISPR nucleases (Cpf1 or Cas9). The primary cells of humanized mice treated with the monodispersed Au NP/crRNA nanoconjugates demonstrated better engraftment kinetics compared with the untreated cells without any significant difference in differentiation [156]. Further studies are required to generate the Au NPs that can perform targeted delivery of the CRISPR/Cas9 system into the cells of interest in vivo.

#### Extracellular Vehicles (EVs)

EVs are cell-derived membranous structures that serve as carriers for the delivery of various types of therapeutic cargoes, such as proteins, lipids, effector molecules, and receptors, to the target cells [180]. EVs are generated through cellular activation or stress. EVs serve as communicating agents between the cells by transporting the contents and surface proteins of the parent to the target cells. Various diagnostic and discovery applications have been demonstrated for EVs. Moreover, EVs are considered promising carriers for safe and robust cell and gene therapy applications that require strong target specificities [181]. In recent years, many studies have reported highly efficient delivery of CRISPR/Cas9 RNPs both in vitro and in vivo via different types of EVs [154, 182, 183]. These studies demonstrated the capability of EVs to be used in clinical settings. EVs are categorized into microvesicles, apoptotic bodies, and exosomes based on their intracellular origins. Among these, exosomes have attracted great interest to be used as carriers for delivery purposes [184]. Exosomes are small membrane-bound EVs (30–150 nm) secreted by the cells through the endosomal route. Exosomes may contain a complex cargo of contents associated with pathological and biological situations of the original cell. Owing to the advantages of exosomes, like inherent non-immunogenicity, these therapeutic carriers have been preferred over the other nano-sized delivery carriers, like polymer-based and Au NPs as well as liposomes [185].

In a recent study, Lin and colleagues developed exosome-liposome hybrid nanocarriers to surmount the inherent limitation of exosomes in the encapsulation of large nucleic acids [158]. Unlike exosomes, these hybrid nanocarriers could successfully encapsulate large-sized plasmids like CRISPR/Cas9 expressing vectors. These hybrid nanocarriers can enter the cells through the endocytosis pathway and induce the expression of the encapsulated genes in the mesenchymal stem cells that were not efficiently transfected with liposomes. Since the exosome–liposome hybrid NPs were able to efficiently deliver CRISPR/Cas9 into mesenchymal stem cells, they can be new candidates for in vivo gene therapy applications which remain open for further investigations.

In another investigation, a new EV-based delivery technology called NanoMEDIC was developed by employing two different homing mechanisms to package CRISPR/Cas9 RNP complex. In this delivery system, Cas9 protein was recruited to extracellular nanovesicles through ligand-dependent dimerization. Next, the two self-cleaving riboswitches and a viral RNA packaging signal held and released sgRNA into these nanovesicles. The NanoMEDIC technology demonstrated >90% efficiency in exon 45 skipping in the *dystrophin* gene of skeletal muscle cells that were derived from iPSCs of a patient with Duchenne muscular dystrophy. Moreover, the muscular injection of these nanovesicles in *mdx* mice and a luciferase reporter mouse model successfully induced transient genomic exon 23 skipping in the *dystrophin* gene [154]. These findings suggest the potential in vivo application of NanoMEDIC for in vivo gene therapy of Duchenne muscular dystrophy and other inherited genetic diseases.

#### Viral Vectors

Viral vectors are common carrier dependent delivery approaches used for gene editing purposes. Among these, adeno, adeno-associated, and lentiviral vectors have been extensively employed for the efficient loading of genetic materials to the target cells in both preclinical and clinical studies. These vectors act as highly efficient in vitro delivery approaches of genome-editing tools for both clinical and research applications [186]. Further details on the viral-mediated CRISPR/Cas9 delivery into different types of stem cells are presented in the following sections, and a summary of these studies is provided in Table 3.

**Table 3:** Viral-mediated delivery of CRISPR/Cas9 gene editing system

Delivery approach	Stem cell type	DNA repair	CRISPR/Cas9 format	Donor format	Editing efficiency (%)	Reference
Adenovirus	HSPCs	NHEJ	HDAd5/35^++^	–	N/A	[126, 187]
		HDR	RNP	HDAd5/35^++^	N/A	[68]
AAV9	Muscle stem cells	NHEJ	Plasmid DNA	–	N/A	[188]
AAV6	HSPCs	HDR	RNP	AAV6	29–69%	[8, 68, 189–194]
	iPSCs/ ESCs	HDR	RNP	AAV6	Up to 94%	[195]
Lentiviral Vector	iPSCs	HDR	Plasmid DNA	LV	N/A	[196]
			RNP	LV	N/A	[197]
	HSPCs	NHEJ	Plasmid DNA	–	N/A	[198]
			mRNA	–	90%	[45]
		HDR	Cas9 protein	IDLV	Up to 42%	[199]
			mRNA	IDLV	20%	[200]
			Plasmid DNA	LV	N/A	[201]
	PSCs	NHEJ	Plasmid DNA	–	N/A	[202]
	ESCs	CRISPRa	Plasmid DNA	–	N/A	[203]
	MSCs	CRISPR/dCas9	Plasmid DNA	–	N/A	[204]

##### Adenoviral Vectors (AdVs)

The AdVs are double-stranded DNA viruses with icosahedral nucleocapsids that are able to infect dividing and non-dividing cells. Owing to their advantages, AdVs are considered promising gene transfer vectors with high transduction efficiency and expression level of the transgene in mammalian cells. Upon injection, the AdVs genome remains extrachromosomal without integration into the host genome [205]. This is especially important for CRISPR/Cas9-mediated genome editing as it minimizes the possibility of insertional mutagenesis and off-target effects. Recently, significant efforts have been made to optimize AdVs for gene delivery applications. In the first attempt, recombinant AdVs were generated by the deletion of the E1 viral gene [206]. Next, a second generation of AdVs with 8 kb packaging capacity was developed through the deletion of E2 and E4 viral genes with the aim of decreasing the chronic immune responses [207, 208]. In the third generation of AdVs, coined as helper-dependent (HDAd) or gutless (GLAd), all the adenoviral genes were deleted to provide a higher packaging capacity of up to 37 kb for loading larger cargoes. These AdVs only contain inverted terminal repeat repeats and packaging signal (ѱ) required for DNA replication and encapsidation, respectively. These unique features make the HDAds an ideal option for encapsulating the CRISPR/Cas9 system in a single vector providing the benefit of delivery procedure simplicity [209, 210]. CRISPR/Cas9 delivery by AdV vectors has already been utilized in drug discovery, disease modeling, and treatments [118]. In 2018, non-integrating chimeric HDAds (Ad5/35^++^) were developed from serotype 5 of species C and serotype 35 of species B AdVs, respectively. The generated vectors interacted with CD46, a membrane protein constantly expressed on human CD34^+^ cells, resulting in efficient transduction of HSCs followed by high expression of CRISPR/Cas9 plasmid. The HDAds (Ad5/35^++^) vector included a number of mutations in the Ad35 fiber knob enhancing CD46 targeting (>25-fold), leading to highly efficient transduction at lower numbers of the multiplicity of infection for viral particles. These chimeric HDAdVs were used to reactivate the fetal γ-globin gene in sickle cell disease and β-thalassemia, affecting the viability, in vitro expansion, and differentiation of human CD34^+^ cells. To control the CRISPR/Cas9 activity, Li and colleagues generated chimeric vectors encapsulating anti-CRISPR (Acr) AcrII4 and AcrII2 peptides to target CRISPR/Cas9 complex (HDAd-Acr). The CD34^+^ cells that were consecutively loaded with HDAd-CRISPR and HDAd-Acr demonstrated a significantly higher engraftment rate. 10 weeks upon transplantation, the engrafted CD34^+^ cells had target site disruption frequencies similar to those of the pre-transplanted cells, demonstrating high viability and good survival of the genetically edited primitive HSCs [126]. Although AdV-mediated gene delivery does not initiate chronic immune responses, the viral capsid still has the chance of inducing acute phase immune responses. It is noteworthy that almost all humans have experienced the AdV infection since infancy, resulting in the production of neutralizing antibodies. Therefore immunogenicity is one of the challenges associated with using AdVs for gene therapy in a variety of human diseases [205, 211]. Different strategies have been proposed to overcome this challenge including manipulation of the vector genome with the aim of decreasing the immunogenicity and chemical protection to reduce the undesirable surface interactions [212].

##### Adeno-Associated Viral Vectors (AAVs)

AAVs, also known as non-enveloped viruses, are among the most popular viral vectors for CRISPR/Cas9-based gene editing purposes. Owing to the unique characteristics of AAVs, such as their good safety profile and therapeutic potential, they have been extensively utilized in gene therapy clinical trials [213]. Other advantages include mild immunogenicity and cytotoxicity observed at high doses of AAVs injection in animal models [213, 214]. Moreover, upon transduction, the AAVs genome usually remains episomal or extrachromosomal, which further integrates into hotspots of mitochondrial DNA and specific locus of the host genome at chromosome 19q13.4 called AAV integration site 1 (AAVS1) [215, 216]. These integrating sites are known as safe harbors without any contribution to tumorigenesis.

Furthermore, AAVs concameters have shown the ability to provide steady transgene expressions due to their long-lasting existence in non-dividing cells. Various AAV serotypes have been shown to be suitable for tissue-targeted gene delivery as well as CRISPR/Cas9-based genome editing in specific tissues. To this aim, AAV6 and AAV9 serotypes have been used for genome editing in murine muscle and brain tissue, respectively [118]. In a study, Xu et al. leveraged the site-specific integration of AAVs to induce long-lasting expression of human blood-coagulating factor IX in transgenic mice [217]. In a recent study, Martin and colleagues proposed a new marker-free approach for genome editing of human pluripotent stem cells (hPSCs) through electroporation of CRISPR/Cas9 RNPs followed by AAV6-mediated donor template delivery. Using this approach, they achieved up to 94% mono-allelic edition frequency of sickle cell mutation at the hemoglobin beta (HBB) site in the hPSCs without requiring marker selection [195]*. *Despite the popularity of AAVs in gene editing clinical trials, the major drawback attributed to these vectors is their limited cloning capacity. Further investigations can focus on the generation of recombinant AAVs with a higher packaging capacity of transgene than the wild type to attenuate the impact of AAVs in clinical gene therapy.

##### Lentiviral Vectors (LVs)

LVs are considered highly efficient vectors for CRISPR/Cas9 delivery into a wide range of dividing and non-dividing cells for gene therapy of monogenic disorders. These vectors can provide the following advantages: i: higher capacity for packaging the transgene, ii) efficient transduction of a different type of dividing and non-dividing cells, iii) low immunogenicity and cytotoxicity, and iv) minimal effect on the cells cycle. These favorable aspects have made LVs the vector of choice for gene-editing of infections associated with hepatitis B virus (HBV), human immunodeficiency virus (HIV-1), and herpes simplex virus (HSV-1), as well as correcting the defects in human genetic disorders like cystic fibrosis [218–220]. Despite these appealing features, LVs suffer from issues such as off-target effects. Since LV-mediated delivery can result in stable expression of the CRISPR/Cas9, it may increase the chance of non-specific DSBs, unwanted off-target effects, and higher indels at off-target sites hindering their application for precise genome editing purposes [220]. To address these issues, integrase-deficient LVs (IDLVs) have been developed for efficient CRISPR/Cas9 delivery with superior cloning capacity while demonstrating a very weak integration capability and transient expression in the host cell [220]. In 2021, an IDLV-mediated CRISPR/Cas9 gene editing approach, coined as an “all-in-one” delivery system, was developed to encode guide RNA and donor DNA templates. Using this strategy, one-time correction of sickle cell disease mutation in the HBB gene was successfully achieved with an efficiency of up to 42% [199]. The application of the IDLVs for CRISPR/Cas9 delivery into the HSCs is yet to be extensively explored.

#### Virus-Like Particles (VLPs)

Although viral vectors have shown success in the efficient delivery of the CRISPR/Cas9 system into the desired cells, major weaknesses attributed to these vectors are the i) high expression level of Cas9 nuclease, ii) increased chance of off-target effects, and iii) immune and inflammatory response issues, and iv) integration into the host genome. Recently, a new type of delivery particle, virus-like particles (VLPs), has been developed for gene editing purposes [221]. These particles are derived from viruses and mimic them in size and shape. The VLPs contain almost all the viral components (e.g., capsid and envelope) except the genome, which eliminates the risk of genome integration and infection in the host cells [222]. These particles are mostly derived from LVs and can package different payloads, including mRNAs, proteins, and RNPs. VLPs take advantage of the high infecting efficiency of viral vectors for transient expression of the Cas9 nuclease resulting in a safe and highly efficient genome editing procedure [221, 222].

Recently, a new type of vesicle, called VEsiCas, was developed to deliver CRISPR/Cas9 RNPs into iPSCs effectively. These VLPs were derived from vesicular stomatitis viruses (VSV) and decorated with fusogenic glycoprotein (VSV-G) for efficient protein delivery. Since VSV-G-enveloped SpCas9 vesicles are free from viral DNA encoding sgRNA and SpCas9, it makes it possible to rapidly clear the nuclease components of the target cells that correlate with reduced genome-wide off-target cleavages. It is shown that in comparison with the classical method of SpCas9 RNPs electroporation for obtaining the transient SpCas9 activities, VEsiCas have lower toxic effects and higher efficiency in nuclease delivery [159].

A follow-up study has reported on the development of engineered murine leukemia VLPs loaded with Cas9-sgRNA RNPs (Nanoblades) for effective genome-editing in primary cells and cell lines such as human hematopoietic and human induced pluripotent stem cells [157]. Moreover, in vivo genome-editing was achieved by transgene-free Nanoblades in the liver of the injected mice and the mouse embryos. The complex of Nanoblades with donor DNA can also be used for homology-directed repair or may be programmed with modified variants of Cas9 for mediating transcriptional gene up-regulations. These engineered VLPs can be easily prepared and are affordable and easy-to-implement in cellular biology laboratories. Since Cas9-sgRNA complex delivery with these VLPs is transient and dose-dependent, it can reduce off-target effects compared with the commonly used CRISPR plasmid transfection method [157].

## Challenges and Future Directions

Despite the advantages of exploiting the CRISPR/Cas9 system for gene editing purposes, extensive research is still required to determine the safety and editing efficiency achieved through this system. The main drawback associated with CRISPR/Cas9-mediated gene editing is the cleavage of off-target genomic sites, as a shorter sequence of the target RNA is exploited in this approach compared to those used in ZFN- and TALEN-mediated gene editing [223]. Although different strategies have been employed to increase the specificity of CRISPR/Cas9-mediated gene editing, such as advancements in gRNA design [224], the development of new versions of the Cas9 nuclease [225], and optimization of the CRISPR/Cas9 delivery mechanisms, off-target effects remain an important hinder to the clinical translation of CRISPR/Cas9-mediated gene editing. In addition, unintentional large deletions and complex genomic rearrangements have been observed in the CRISPR/Cas9 edited cells [226]. On the other hand, inherent individual human genetic variations (e.g., single nucleotide polymorphisms and copy number variations) can lead to unintentional off-target gene editing. Although the standard human genome is often used as a reference for CRISPR/Cas9 optimization and off-target tool design, these polymorphisms can lead to multiple off-targets even using well-designed gRNAs [227]. Therefore, genome-wide sequence analysis, large-scale off-target prediction, individual intensive genotoxicity risk assessment, and careful patient monitoring are the measures to be considered in CRISPR/Cas9-mediated gene editing. The immunogenicity of the Cas9 nuclease also needs to be considered during the clinical translation of the CRISPR/Cas9 technology, which can cause severe immune responses in patients treated with CRISPR/Cas9 edited stem cells [228].

### Base-Editing and Prime-Editing as Novel Gene Editing Approaches

The CRISPR/Cas9-mediated genome editing relies on the induction of a DNA double-strand break in the target DNA sequence. The generation of these breaks raises several concerns about the clinical applications of this gene editing strategy. DNA base-editing and prime-editing are novel gene editing approaches proposed to overcome the field’s current challenges. DNA base-editor is an engineered Cas enzyme that can bind to the target site and modify the target nucleotide without generating DSBs. Adenine base-editor (ABE) and cytosine base-editor (CBE) are two well-known types of DNA base-editor systems. Until recently, the correction of four transition mutations (C → T, G → A, A → G, and T → C) was only possible using the known CRISPR/Cas9 base editing. A recent study has proposed two new base editors for efficient C to G transversion [229]. In addition, dual base-editor Cas enzymes have been engineered recently for combinatorial editing in human cells [73, 230, 231]. Taken together, the engineered base editors broaden the application of DNA base-editing to transversion mutations and more complex edits that are impossible using single DNA base-editors.

Prime-editing is another gene editing strategy recently developed to expand the range of mutations that can be edited [232]. Unlike CRISPR-based editing, prime-editing does not require double-strand DNA breaks. The prime-editing system consists of a Cas9 H840A nickase fused to an engineered reverse transcriptase enzyme and a prime editing gRNA (pegRNA). The pegRNA is an extended guide RNA that includes a primer binding site (PBS) and a reverse transcriptase (RT) template sequence, which is then reverse-transcribed to DNA by the RT enzyme. Prime editing has several benefits over CRISPR/Cas9-mediated gene editing, including the ability to introduce nearly all conceivable nucleotide substitutions, the absence of a need for simultaneous delivery of a corrective donor template, the elimination of indel-induced frameshifts, and a lower rate of off-target edits. Consequently, it is a new and ideal candidate for overcoming the limitations of current CRISPR/Cas9- mediated gene editing, which can open up promising avenues toward more versatile and improved genome editing [233].

## Conclusion

Different factors can affect the efficiency of CRISPR/Cas9-mediated gene editing, and the delivery approach is an essential factor that plays a vital role in the efficient gene editing process, especially in the case of hard-to-transfect stem cells. Since gene editing in stem cells can translate to the clinic, developing effective methods for delivering gene-editing components to different types of stem cells is necessary. This review paper entails a wide range of physical, chemical, mechanical, viral, and nanoparticle-based methods used to deliver gene editing tools to stem cells. These methods categorize into two main classes, carrier-dependent and carrier independent delivery approaches. Amongst them, a subset of carrier independent delivery approaches, called physical delivery (e.g., electroporation), is the most widely used method that gained much more attention for delivery applications. Several efforts have been made to enhance the efficiency of loading gene editing tools into target cells by combining some of the aforementioned methods. Since gene editing approaches are evolving rapidly, choosing an effective mechanism for loading the newly developed gene editing tools into the target cells among the existing options relies on the aims and objectives of the experiments. Developing novel delivery technologies that efficiently deliver gene editing tools inside the target cells without affecting the cell functionality will significantly impact the research and clinical outcomes of stem cell gene editing with remarkable accuracy.

## Data Availability

This is a narrative review based on published data.

## Abbreviations

*CRISPR/Cas9*: clustered regularly interspaced short palindromic repeats/CRISPR-associated protein 9
*hPSCs*: human pluripotent stem cells, indel: insertion or deletion
*DSBs*: double-strand breaks
*NHEJ*: non-homologous end-joining
*HDR*: homology-directed repair
*TALENs*: transcription activator-like effector nucleases
*ZFNs*: zinc-finger nucleases
*sgRNA*: single guide RNA
*crRNA*: CRISPR RNA
*tracrRNA*: trans-activating crRNA
*RNP*: ribonucleoprotein
*iTOP*: induced transduction by osmocytosis and propanebetaine
*iPSCs*: induced pluripotent stem cells
*DDEB*: dominant dystrophic epidermolysis bullosa
*RDEB*: recessive dystrophic epidermolysis bullosa
*ssODN*: single-stranded oligodeoxynucleotides
*ABE*: adenine based editor
*PSCs*: pluripotent stem cells
*HSCs*: hematopoietic stem cells
*CBE*: cytosine base editor
*NB-Chip*: nano-blade chip
*TRIAMF*: transmembrane internalization assisted by membrane filtration
*NPs*: nanoparticles
*LNPs*: lipid-based NPs
*FDA*: food and drug administration
*BrS*: Brugada syndrome
*PLGA*: poly lactic-co-glycolic acid
*EMA*: European medicine agency
*HSPCs*: hematopoietic stem and progenitor cells
*EVs*: extracellular vesicles
*AdVs*: adenoviral vectors
*HDAd*: helper-dependent adenovirus
*GLAd*: gutless adenovirus
*Acr*: anti-CRISPR
*AAVs*: adeno-associated viruses
*AAVS1*: AAV integration site 1
*HBB*: hemoglobin beta
*LVs*: lentiviruses
*HBV*: hepatitis B virus
*HIV-1*: human immunodeficiency virus-1
*HSV-1*: herpes simplex virus
*IDLVs*: integrase-deficient LVs
*CRISPRa*: CRISPR activator
*CRISPR/dCas9-E*: CRISPR/dcas9-effector
*VLPs*: virus-like particles
*VSVs*: vesicular stomatitis viruses
*VSV-G*: vesicular stomatitis viruses decorated with fusogenic glycoprotein
*pegRNA*: prime editing gRNA
*gRNA*: guide RNA
*PBS*: primer binding site
*RT*: reverse transcriptase
